# A Comparative Study of Physiological Monitoring with a Wearable Opto-Electronic Patch Sensor (OEPS) for Motion Reduction

**DOI:** 10.3390/bios5020288

**Published:** 2015-06-08

**Authors:** Abdullah Alzahrani, Sijung Hu, Vicente Azorin-Peris

**Affiliations:** School of Electronic, Electrical and Systems Engineering, Loughborough University, Ashby Road, Loughborough, Leicestershire LE11 3TU, UK; E-Mails: A.Alzahrani@lboro.ac.uk (A.A.); V.Azorin-Peris@lboro.ac.uk (V.A.-P.)

**Keywords:** Accelerometer, Adaptive filter, Artefact motion, photoplethysmography, real-time physiological monitoring, Opto-Electronic Patch Sensor (OEPS), personal healthcare

## Abstract

This paper presents a comparative study in physiological monitoring between a wearable opto-electronic patch sensor (OEPS) comprising a three-axis Microelectromechanical systems (MEMs) accelerometer (3MA) and commercial devices. The study aims to effectively capture critical physiological parameters, for instance, oxygen saturation, heart rate, respiration rate and heart rate variability, as extracted from the pulsatile waveforms captured by OEPS against motion artefacts when using the commercial probe. The protocol involved 16 healthy subjects and was designed to test the features of OEPS, with emphasis on the effective reduction of motion artefacts through the utilization of a 3MA as a movement reference. The results show significant agreement between the heart rates from the reference measurements and the recovered signals. Significance of standard deviation and error of mean yield values of 2.27 and 0.65 beats per minute, respectively; and a high correlation (0.97) between the results of the commercial sensor and OEPS. T, Wilcoxon and Bland-Altman with 95% limit of agreement tests were also applied in the comparison of heart rates extracted from these sensors, yielding a mean difference (MD: 0.08). The outcome of the present work incites the prospects of OEPS on physiological monitoring during physical activities.

## 1. Introduction

In recent decades, the number of people suffering from chronic diseases has increased dramatically. Cardiovascular diseases are the number one cause of death globally, with more people dying annually from these than any other cause [1]. Almost 2.6 million people in the UK are suffering from heart and circulatory diseases [2]. In today’s growing and ageing population, cardiovascular disease, stroke and diabetes are the main causes of disability and death [3]. Unfortunately, the number of people dying from these kinds of diseases has increased in recent years. This brings increasing demands for personal healthcare solutions to provide continuous and cost-effective observation of physiological parameters, to diagnose health conditions early, towards a more effective prevention and management of such illnesses. Telemedicine technology is an important trend in remote healthcare monitoring which is well developed and is becoming a valuable means to reduce the costs of treatment and to increase service quality in the healthcare sector. As indicated by WHO [4], continuous monitoring is an effective means not only to assess physiological status related to these diseases, but also to provide indications of disease progression and to make it easier to manage the day-to-day procurement of healthcare. It is, therefore, of vital importance to develop a cost-effective means to minimize the onset and consequences of these diseases.

Opto-physiological modelling (OPM) driven Photoplethysmography (PPG) [5], is a non-invasive optical technique that is used to measure dynamic changes in tissue optical properties, such as blood volume change in a micro-vascular bed of tissue. The effective monitoring and assessment of vital signs using PPG-based techniques is reliant on an understanding of the opto-physiological interaction between illumination and biological tissue. Equation 1 is a formulation of Lambert Beer’s law, where source light of intensity *I_0_* travels a path length *d* through a body of pulsatile tissue characterized by an absorption coefficient, $\eta_{eff}$, yielding transmitted (detectable) light of intensity *I*.

(1) $$I=I_{0}.e^{-(d.\eta_{eff})}$$

PPG-based techniques can provide valuable information about physiological status, such as heart rate, blood pressure, oxygen saturation (SpO_2_%), respiration rate and heart rate variability (HRV), where the latter yields information about the autonomic nervous system (ANS) [6].

The issue of noise artefacts in PPG techniques is presented as one of greatest challenges to overcome, and there have been attempts to minimize these kinds of artefacts by using a variety of approaches. The work conducted by Gibbs [7] introduced an adaptive noise cancellation [8,9]. Correlation [10], spectral [11], and non-linear [12] cancellation are some of the most common methods used to enhance pulsatile signals through the suppression of artefacts. Another approach to compensate for artefacts in PPG signals involves recognition of these artefacts, either through feature-based recognition of corrupt pulses [13] or through identification of interpretation errors [14].

A number of attempts have been made towards finding a reliable approach to the reduction of this type of artefact, ranging from using hardware, with active or passive filtering, to various signal processing methods. The hardware method failed when artefacts present within the dominant range of PPG signals, thus, making it difficult to distinguish between useful physiological data and artefacts. Most of signal prosing approaches lack generality, as they cannot recover a desirable signal across a wide range of different scenarios [15].

As expressed in [16], physiological parameters, such as oxygen saturation (SpO_2_%), heart rate, respiration rate and heart rate variability, are strongly related to physical activities that can be tracked by an accelerometer. A study [17] involving an accelerometer and PPG measurements has shown that PPG signals could be recovered in the presence of mild motion artefacts, where a zero-phase digital filtering was engaged to reduce inaccuracy on the PPG signals in states of poor perfusion.

Another approach is independent component analysis (ICA) as indicated by Yoo *et al*. [18] recommended using a block interleaving and basic ICA algorithm. Natarajan *et al.* [19] proposed using ICA in the frequency-domain. The ICA approach assumes that the subcomponents are non-Gaussian signals and that they are statistically independent from each other, thus the ICA does not yield good separation in PPG signals contaminated by motion artefacts, as suggested by Yao and Warren [20]. The periodogram algorithm method was been used to estimate the heart rate (HR), nevertheless, this technique has some drawbacks as due to inconsistent spectral estimates, high variance as well as serious leakage effects [21]. Adaptive noise cancelation (ANC) is also proposed as a technique that can help to remove or eliminate motion artefacts, as Ram *et al*. [22] and Yousefi *et al.* [23] suggested. Most of the processing techniques were proposed for specific scenarios and require careful setup, when users performed detailed instructions and small motion, such as keyboard typing, finger movements [21,22] and walking [23].

Among previous approaches and methods, acceleration data (AD) has been shown to assist in the reduction of motion artefact. Fukushima *et al.* [24] suggested a spectrum subtraction method to cancel the spectrum of acceleration data from the desired PPG signals. However, a lack of generality is imposed by the implicit assumption that artefact corruption manifests itself as an additional signal component unrelated to physiology either in time, frequency or statistical domains [20]. The AD with Kalman filtering (KF) as indicated by Lee *et al.* [25] can be used to reconstruct the signals from motion artefacts but is computationally intensive. Asada [26] used a micro accelerometer and Laguerre series adaptive filters, but the experimentation and simulation showed that, while the proposed method worked well at certain times, the results were not reliable during jogging. The detection of physical activity could provide a reference to be used to recover signals corrupted by body movement [27]. These artefacts could also be reduced through an appropriate engineering approach that combines an optimum set of features, such as the right hardware to facilitate algorithms for self-cancellation of noise, and an optical sensor allocation to enhance the fundamental stability of the signals.

In this study, an analogue three-axis MEMS accelerometer (3MA) was employed to detect acceleration and movement in all three axes with respect to gravitational acceleration and to provide a reliable movement reference. The 3MA provides a high accuracy in the characterization of physical movement with its three signal outputs for accelerations along spatial vectors X, Y and Z. The value of 3MA stems from the fact that capacitance changes inside the accelerometer reflect forces on all three axes in respect of gravitational acceleration. The total acceleration vector is produced as a combination of vectors X, Y and Z. When the user’s body moves, the accelerometer produces signals proportional to the magnitude of the movement, and the resultant total acceleration vector represents the instantaneous direction and magnitude of motion in 3D space.

The work presents an effective way to capture critical physiological parameters through a wearable OEPS comprising a 3MA and real-time wireless communication to a host. The objectives of this study are: (1) to attain a better understanding of the interaction between illumination and biological tissue in order to provide continuous and accurate health assessment; (2) to test physical activity monitoring when using motion characterization for recovery of signals corrupted by body movement; and (3) to move towards an appropriate electronic design and development of algorithms for self-cancellation of noise and increased stability of the opto-electronic patch sensor (OEPS).

## 2. Method and Experimental Setups

### 2.1. Construction of Opto-Electronic Patch Sensor (OEPS)

**Figure 1:**
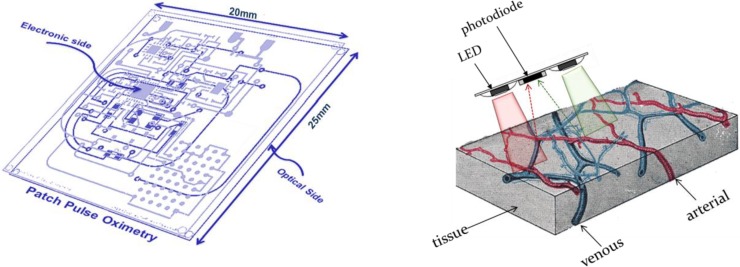
Overview of the wearable OEPS system design along with initial prototype.

**Figure 2:**
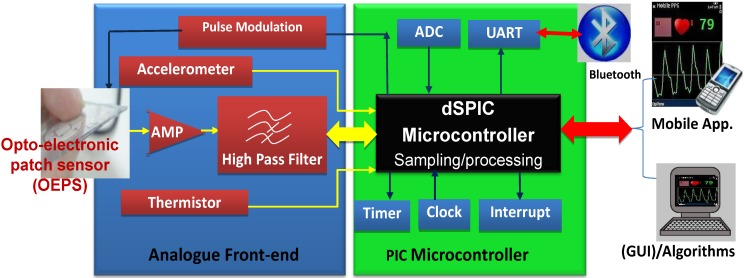
Overview of OEPS electronic system for continuous physiological monitoring.

The OEPS is a miniaturized and wearable device [28]. It was constructed for continuous measurement of blood volume changes in the vessels, corresponding to pulsations of the heart. The OEPS operates in reflectance mode (light source and detector are on the same surface) and is suitable for being attached or adhered to different locations on the human body, for instance, forehead, palm, earlobe and wrist as shown in Figure 1. The schematic of the OEPS consisting of an opto-electronic sensor, a 3MA and a body temperature sensor, as in presented Figure 2, shows all integrated within one piece of electronic printed circuit board (PCB). The opto-electronic sensor uses multi-wavelength illumination sources, including, green 525 nm, red 660 nm and infrared (IR) 990 nm (JMSienna Co., Ltd), and a low-profile photodiode (BPW34SR18R, Osram, GmbH).

Figure 2 shows the system as a whole, consisting of: (1) A customized wearable sensor comprising opto-electronics to capture physiological signals, an analogue three-axis MEMS accelerometer (3MA, ADXL337, Analog Devices Inc., Norwood, MA., USA) to characterize motion, a body temperature sensor (SMD3 0402, Measurement Specialties), and a suitable analogue front-end with pre-amplification and high-pass filtering; (2) a PIC Microcontroller (Microchip Technology Inc., Chandler, AZ., USA) to implement low pass filtering, sampling of signals (ADC) and to process the captured signals for preparation of data for wireless transfer to a smart-phone via a Bluetooth module (UART protocol); and (3) mobile device or Bluetooth-enabled computer to receive the data, to optionally process the desired signals further, and finally to display the available data in a suitable graphical form.

### 2.2. Principle of Motion Artefact Reduction 

The 3MA acceleration output is measured in three dimensions (in the form of *X, Y* and *Z* vectors), which are then integrated to represent movement over a certain time using a square root of the sum of squares of all three vectors. The vector *R* is a force vector that represents the movement as can be measured through the projection of the combination of *Rx*, *Ry* and *Rz*. The integrated signal for movement over time can be represented by vector magnitude units (VMU) [29] using a process known as acceleration synthesisation.

(2) $$VMU\;\left(R\right)=\;\sqrt{Rx^{2}+Ry^{2}+Rz^{2}}$$

The values of projection appearing in the corrupted PPG are closely related to the values produced from the 3MA. The analogue output of 3MA can be converted into a voltage level through the ADC channel of the microcontroller (Microchip Technology Inc.). The vector magnitude of acceleration is mainly linear when a subject moves along a specific axis, or when the movement is periodically consistent. In a real situation of measurement, the resultant acceleration could not be linear since the physical movement is not often regular. The approach of acceleration decomposition is a 3D expression method as the features of 3MA are in some arbitrary orientation on the wearer’s body [30]. The algorithm works on a period of sampling intervals chosen to estimate a vertical acceleration vector on each axis corresponding to the gravity component, then derived from the averages of all axes on the same sampling interval.

(3) AV=(AVx, AVy, AVz)

Here, *AV_x_, AV_y_* and *AV_z_* are the average vertical components of gravity in each axis.

The processing data holds the vector of acceleration on each axis at a given point in the same sampling interval and can be represented as in Equation (4):

(4) V=(Vx, Vy, Vz)

Here, *V_x_*, *V_y_* and *V_z_* are the vectors of the three accelerations at a given point in time.

The vector *V* in fact represents the superposition of forces due to gravity and motion of the subject’s body in 3D space. The dynamic component of each axis originating solely from the body motion, disregarding the gravity component, can be expressed as in Equation (5):

(5) d = ( Vx – AVx, Ay – AVy, Az – AVz )

In order to deduce the horizontal component of the dynamic acceleration *H*, the projection *p* or the vector dot products of the dynamic component *d* upon the average vertical axis *AV* can first be computed as in Equation (6):

(6) $$p=\left(\frac{d*AV}{AV*AV}\right)*AV\;$$

The horizontal component of the dynamic acceleration *H* can then be expressed as in Equation (7):

(7) H=d−p

As a consequence, the algorithm operates on a sequence of samples and estimates the magnitude of vertical and horizontal components of the dynamic accelerations, each being independent of the orientation of the object. The processed data of the accelerometer is then mapped with the contribution of the motion artefact that appears in the corrupted signals, followed by extraction of the reduced artefact PPG waveform.

The manifestation of motion artefact is dependent on the direction and nature of movement; for example, if the OEPS is placed in the wrist, it generates different motion artefacts than if it is attached to the forehead. In the present design when a movement takes place, the 3MA produces a signal proportional to the body movement regardless of the direction of the movement and the position of the OEPS, since the system design uses a combination circuit. This helps to understand where there was no need to match the phase of the reference motion signals. This study introduced a cancellation method to reduce motion artefacts on corrupted PPG signals using the reference signals from the 3MA, followed by an adaptive filter to facilitate the optimum adaptive motion artefact cancellation technique.

### 2.3. Adaptive Motion Artefact Cancellation

**Figure 3:**
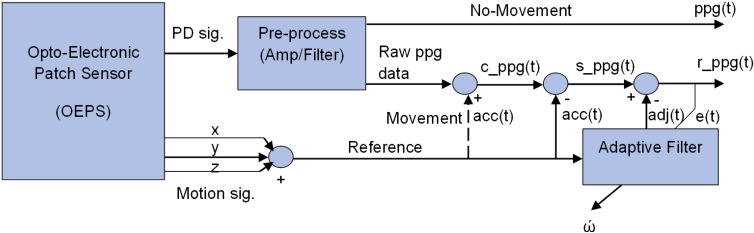
System schematic of adaptive motion artefact cancellation technique.

Figure 3 illustrates a method for adaptive motion artefact cancellation (AMC) with a combined correlated and uncorrelated approach. To realize the adaptive motion cancellation, the OEPS system uses two inputs. The method uses the corrupted PPG signal *c_ppg(t)* as a primary input, and the combined motion signal a*cc(t)* from the accelerometer as a reference input, which is assumed to correlate in some intervals with the noise in the primary input. Both signals from the 3MA and the OEPS are digitized in the microcontroller to perform this kind of processing. In the absence of movement, no signal comes out from the reference accelerometer, and a clean *ppg(t)* signal is passed directly to the output.

To achieve adaptive motion artefact cancellation, the motion signal a*cc(t)* is subtracted from the primary input *c_ppg(t)* to cancel out the motion correlated parts of the latter, and the resultant signal *s_ppg(t)* passes to an adaptive filter that facilities non-linear adjustment of weight by using a least mean square algorithm [31], to yield a recovered output signal *r_ppg(t)*. The adaptive filter enhances the desired part of the signal to produce an output *adj(t)* that is as close as possible to *acc(t)* by readjusting itself automatically and continuously, using variable coefficient *w* to minimize the error between *acc(t)* and *adj(t)* during the process. The noise component in the output signal *r_ppg(t)* is finally minimized by subtracting the output of the filter *adj(t)* from the resultant signal *s_ppg(t)* as shown in Equation (13):

(8) r_ppg(t) = s_ppg−adj(t)

When the OEPS system defines a minimum between *acc(t)* and *adj(t)*, the output signal is regarded as a clean *ppg(t)* signal. To reach this point a better solution to meet the requirement of the *OEPS* system is to uses the least mean squares algorithm (*LMS*) to minimize total system output power. The system output serves the error signal *e(t)* for the adaptive process. The output *e(t)* is represented in Equation (9).

(9) e(t)=ppg(t)+acc(t)−adj(t)×w

Squaring both sides of Equation (9) yields Equation (10):

(10) $$e^{2}\left(t\right)={\left(ppg\left(t\right)+acc\left(t\right)-adj\left(t\right)\times w\right)}^{2}\;e^{2}\left(t\right)=ppg^{2}\left(t\right)-2\times ppg\left(t\right)\times \left(acc\left(t\right)-adj\left(t\right)\times w\right)+{\left(acc\left(t\right)-adj\left(t\right)\times w\right)}^{2}$$

Here, taking the expectations of both sides of Equation (9), and assuming from Figure 3 that the signal that passes to the adaptive filter is no longer correlated with *acc(t)* or with *adj(t)*, yields Equation (11)*:*

(11) $$E[e^{2}\left(t\right)]=E[ppg^{2}\left(t\right)]+E[{\left(acc\left(t\right)-adj\left(t\right)\times w\right)}^{2}]$$

The *ppg* signal power *E*[*ppg^2^*(*t*)] is independent and unaffected as the filter is adjusted to minimize *E*[(*acc*(*t*)*-adj*(*t*)×w)^*2*^]. The filter output *adj*(*t*) is a best least squares estimate of the primary noise *acc*(*t*). Minimizing the total output power *E*[*e^2^*(*t*)] results in minimizing the noise power *E*[(*acc*(*t*)*-adj*(*t*)×w)^2^]. Since the signal of *E* [*ppg^2^*(*t*)] remains separated, minimizing the total output error by power squaring results in the squaring of the desired PPG signal and a maximized signal-to-noise ratio (SNR) in the output.

### 2.4. Experiment Setup and Data Collection

The study was carried out to investigate the performance of the OEPS system under five different types of physical activity: standing, sitting, walking 3.0 to 6.0 km/h, cycling 20.0 to 35.0 km/h and running 7.0–8.5 km/h. Sixteen subjects (15 males, 1 female) between the ages of 20 to 47 years participated in the experimental protocol with approval of the Loughborough University Ethics Committee. Prior to the recordings, each subject’s body mass index (BMI), blood pressure and oxygen saturation were taken, and the room temperature (°C) and humidity (%) were noted.

**Figure 4:**
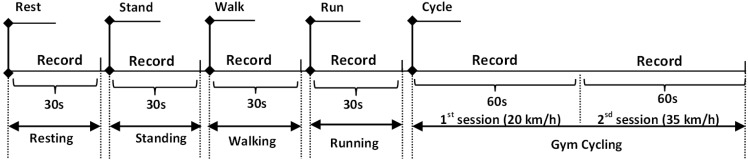
A schematic diagram of physiological monitoring protocol with five different exercises.

Sixteen subjects were divided into four groups, where the subjects in each group had the OEPS placed on a different measuring site*,* namely, palm, forehead, finger or earlobe. Meanwhile a commercial reflectance contact pulse oximetry probe (Nellcor probe, NellcorTM Max-FastTM, COVIDIEN) and a cuff blood pressure meter (Omron blood pressure meter, M6 COMFORT OMRON Inc., Chicago, IL, USA) were placed on the other side of the subject’s body. The 3MA was attached on the back of the OEPS in order to be adjacent to the optical signal-capturing site. The subjects were asked to perform a variety of designated physical activities whilst individual recordings were taken for each of these. Specifically, the recordings were taken whilst the subjects were standing at rest, sitting at rest, walking, running and cycling on the gym cycle. Recordings of 30 s were taken for each individual exercise, with the exception of the cycling exercises, where the data were recorded for a total of 120 s. The increments of cycling exercises were applied in the protocol at the speed from 20 km/h up to 35 km/h: the first recording is 60 s, followed by another 60 s recording at the maximum speed of 35 km/h. Figure 4 shows the protocol of entire exercise procedures as performed by subjects.

Both the OEPS and Nellcor probe were driven by a custom PPG board (DISCO4, Dialog Devices Ltd., Reading, UK), which outputs an analogue PPG signal for each. Analogue-to-digital conversion of all captured signals (Green, Red and IR PPG signals; *X*, *Y*, *Z* motion signals from 3MA) was implemented using an external USB data acquisition board (USB-6009, 14 bits per sample, National Instruments Co., Austin, TX,USA). LabVIEW (National Instruments Co.) was used to implement the front-end applications for control of intensity of the PPG probe light sources, control of USB data acquisition and display of raw, filtered and reference 3MA signals. MATLAB (The MathWorks Inc., Natick, MA, USA) was used to perform the signal processing and analysis necessary to evaluate the performance of the proposed system.

The data from the OEPS, the commercial reflectance contact pulse oximetry probe, the cuff blood pressure meter and the acceleration reference signals from the 3MA were all captured simultaneously in order to facilitate the processing of signals. A sampling frequency of 128 Hz was selected as an adequate rate for representation of the PPG signal, that is, to meet the requirements of the Nyquist frequency.

## 3. Results 

**Figure 5:**
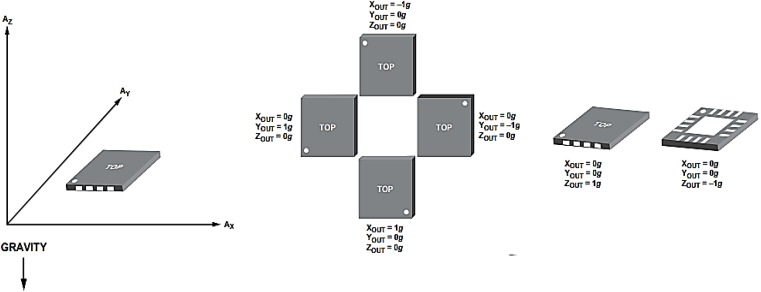
Output response *vs*. orientation to gravity.

In this study, the 3MA was tested and calibrated using an oscilloscope (TDS210, Tektronix Inc., Plano, TX, USA) and multimeter (77IV, Fluke Co., Fluke Co., NH, USA) to present the readings of X-axis, Y-axis and Z-axis with a correct offset setting. For instance, readings of (*x*, *y*, *z*) from the 3MA were taken in three different positions in the absence of movement. The first reading was taken with the 3MA sitting flat on a table, in other words, with 0 *g* gravitational acceleration in the *x* and *y* axes. For the second reading, the 3MA was placed with its x-y plane at 90° to the table. The third reading was taken with the 3MA sitting flat on the table with the opposite face as in the first reading, corresponding to −1 *g* on the *z* axis. Figure 5 shows the output response of the acceleration *versus* gravity.

All three calibrations were taken according to the output response to gravity in each position. Table 1 presents the calibration measurements are close to the offset reading in the datasheet of the accelerometer as is vital to compensate the processing data in the system.

**Table 1:** Calibration of 3MA offset along three axes.

State	Calibration Method	*Vx (v)*	*Vy (v)*	*Vz (v)*
**Acc. On the table**	oscilloscope	1.80	1.60	1.60
	multimeter	1.73	1.48	1.48
**Acc. is vertical**	oscilloscope	1.40	1.85	1.85
	multimeter	1.40	1.62	1.62
**Acc. is reverse vertical**	oscilloscope	1.45	1.42	1.42
	multimeter	1.45	1.30	1.30

**Figure 6:**
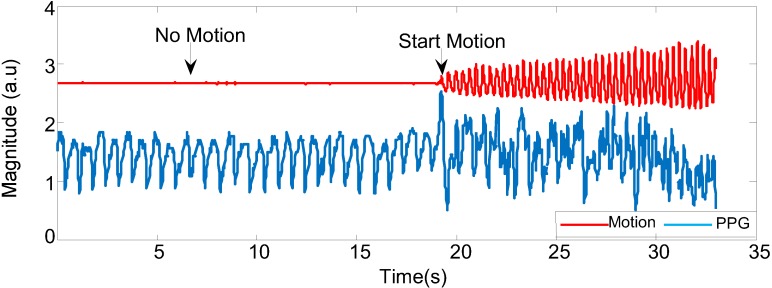
The corrupted PPG signal with different movements.

Figure 6 shows that the acceleration signal of combined three axes *x, y* and *z* and changes in PPG signals while the participant is still and in motion. When the subject is doing any kind of motion (in this case, walking fast), the PPG signal starts to be corrupted; in the absence of motion, the PPG signal is stable.

**Figure 7:**
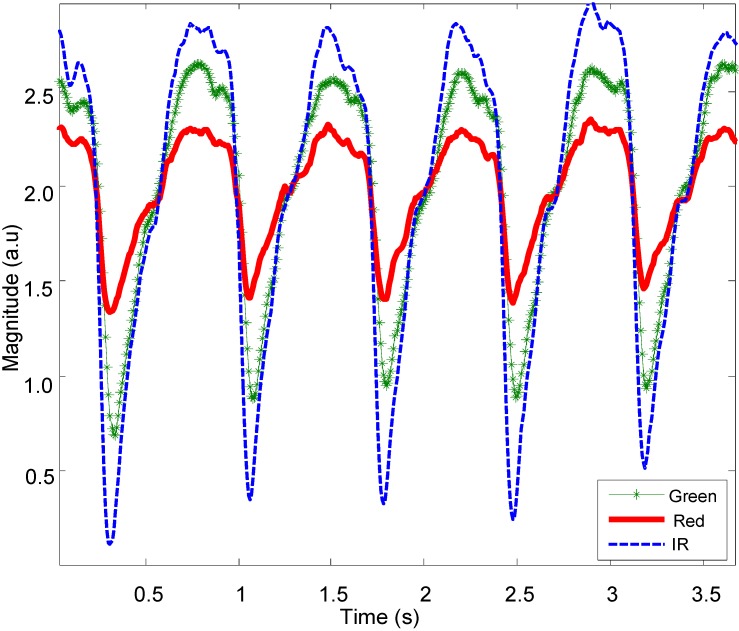
PPG signals captured by OEPS with green (525 nm), red (650 nm) and IR (870 nm) LED illumination.

To evaluate the performance of the proposed motion artefact reduction approach, three characteristics were examined in the captured PPG data: (1) the quality of signal reconstruction via visual inspection; (2) the differences in heart rate between the reference signals and the recovered signals; and (3) the quality of recovered signals via visual inspection of their frequency content. Figure 7 shows representative normal PPG signals from the OEPS at multiple wavelengths as captured from the palm of a subject at rest (sitting).

**Figure 8:**
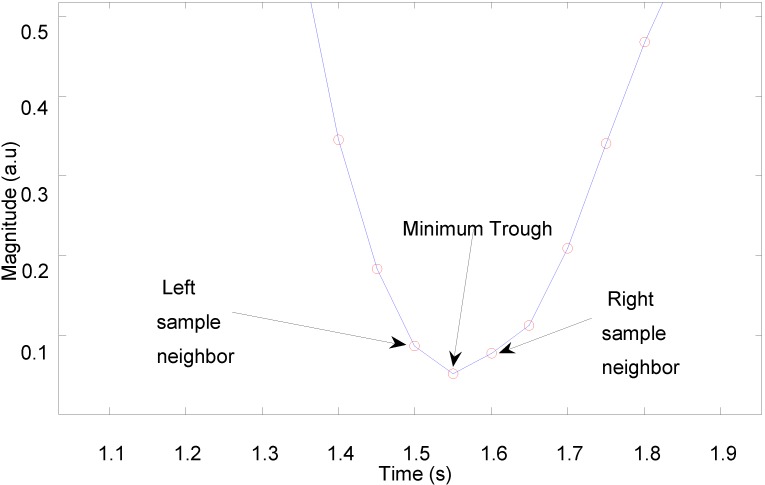
Trough detection of pulsatile waveform.

An algorithm for detection of peaks or troughs in the captured PPG signals was developed to calculate heart rate. Figure 8 shows a trough detection approach as a consistent and easy way to select a lowest point.

The peak or trough detection algorithm sweeps across all samples of a PPG signal in order to determine which samples are smaller (or larger) than its two neighbouring samples. Knowing the temporal distance between two troughs, the pulse-pulse interval is simply calculated as the reciprocal of the distance between the troughs, where the temporal distance is converted to units of minutes to yield an instantaneous heart rate in beats per minute. The algorithm ultimately determines all the pulse intervals within a certain period, and its outputs can subsequently be used to calculate heart rate variability (HRV).

**Figure 9:**
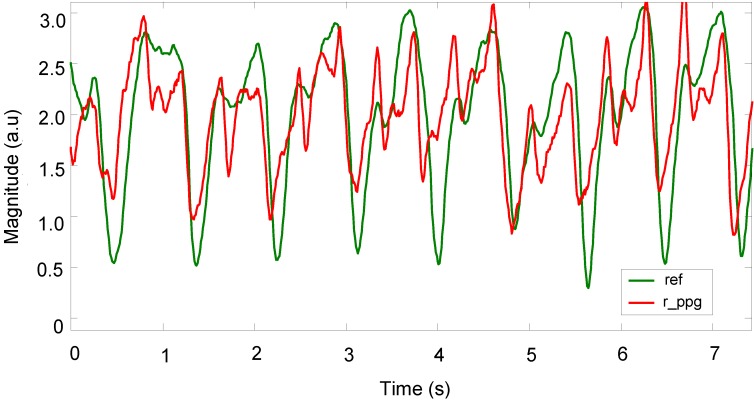
Recoveries of PPG signal after applying vector magnitude.

**Figure 10:**
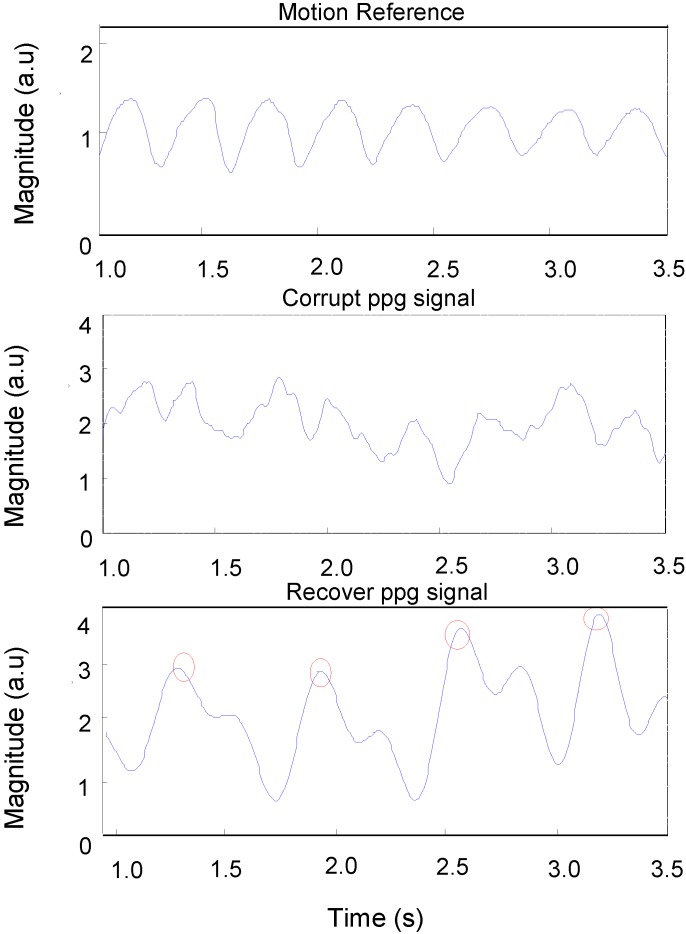
Recovery of PPG signals using automatic adaptive cancellation.

Once the vector magnitude algorithm is used to produce the reference signal, and following the initial noise cancellation stage, the peaks of the signal can be detected, but there are still some uncorrelated values between the prime processed PPG signals and reference 3MA signals. Figure 9 shows a representative PPG signal that was recovered using the 3MA as a reference for motion. Here, the measurement was taken from the palm during fast walking (6 km/h) exercise, with the 3MA attached on the back of the patch sensor. At this stage, the troughs of the signal are visible but the signal still requires further processing. In order to provide a more stable PPG signal, the adaptive filter for reducing motion artefacts was set to a filter order of 32 and a step size of *μ* = 0.1. A Butterworth low-pass filter (16th order, finite impulse response, 5 Hz cut-off) was also used in order to eliminate any further high noise that was out of the frequency range of interest, of 1–5 Hz, as shown in Figure 10.

**Figure 11:**
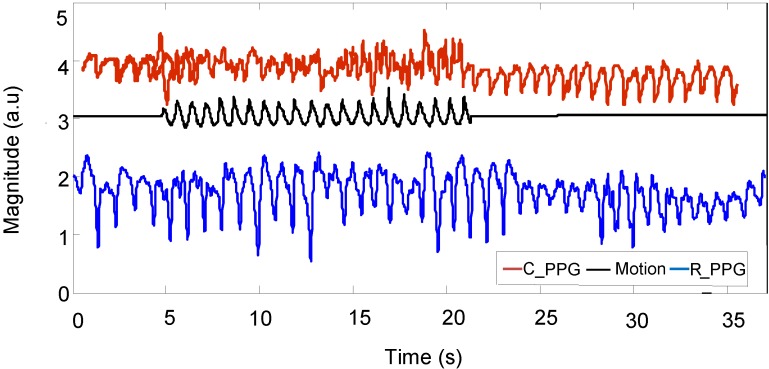
Corrupted PPG signal, motion acceleration and recovered PPG signal.

In order to determine the quality of the reconstruction of signals, the data was visually examined to determine whether a pulsatile waveform was consistent and correlated with the reference signal. Figure 10 shows: (1) a motion reference signal; (2) a corrupted PPG signal captured from a subject’s palm during fast walking exercise; and (3) the same PPG signal after applying automatic adaptive motion artefact cancellation. The pulse-pulse intervals (PPI) can be clearly seen in the recovered PPG signal with the presence of motion artefacts. From this PPI, the heart rate and heart rate variability can be measured. Figure 11 shows a motion acceleration reference, corrupted PPG signal (*C_PPG*) due to running movement and recovered PPG signal (*R_PPG*).

**Figure 12:**
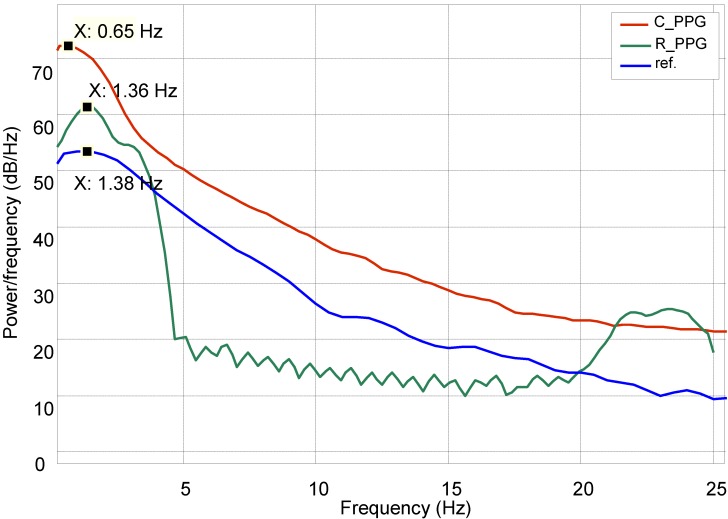
Power spectral density estimation (PSDE) of the PPG.

While processing heart rate variation, the recovered signal was compared with a normal PPG reference in the frequency domain by using power spectral density estimation (PSDE); the result is shown in Figure 12.

Figure 12 shows how the spectral characteristics of a recovered signal (*R_PPG*) are well matched with those of its reference signal (ref), both are in the same dominant frequency (around 1.3 Hz), whereas the spectral characteristics of the original corrupted signal (*C_PPG*) do not match those of its clean reference signal. The comparison shows a slight variation (*σ* = 2%) around the centre dominant frequencies of the reference and recovered signals.

In addition, the algorithm for heart rate detection was executed on subject data acquired during cycling exercise and comparing with reference heart rate measurements taken from the commercial blood pressure monitor, as well as from the commercial reflectance pulse oximetry probe. Table 2 summarizes the measurement of the gym cycle, which includes blood pressure (BP, mmHg), heart rate (HR, bpm), speed as (Km/h) and pedalling rate (RPM, rev/min).

Table 2 shows the heart rate (HR) during cycling is well matched between OEPS and the reference devices. The average heart rate during cycling exercise is close to the heart rate captured from the commercial devices, although a small variation of ±2 bpm is observed. Statistical significance testing was implemented to validate the comparison between these datasets.

**Figure 13:**
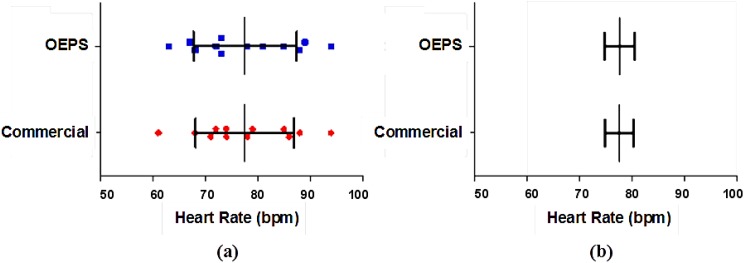
Statistical Significance test of the HR data: (**a**) Distribution of HR data showing mean and SD; (**b**) Mean and SEM of HR data.

Figure 13 shows a *t-test* to determine the variance and correlation of the data. The result shows no significant difference between the heart rate (HR) that was measured from the commercial devices and from the OEPS.

**Table 2:** Measurement of physiological parameters during cycling exercise, 20–35 km/h.

Participants		BP _Sys_	BP _Dia_	H.R.	H.R.	SpO_2_	Speed	R.P.M	H.R.
No.	Age	(mmHg)	(mmHg)	O(bpm)	N(bpm)	(%)	(Km/h)	rev/min)	OEPS(bpm)
1	39	125	93	94	94	98	30	83	94
2	35	123	83	67	68	99	31	87	67
3	37	102	78	70	71	97	30	82	72
4	21	124	71	70	74	95	25	70	68
5	22	132	90	85	85	97	27	71	85
6	43	106	78	87	86	98	29	80	89
7	46	168	114	74	74	98	28	73	73
8	30	115	62	62	61	99	26	67	63
9	24	122	77	80	79	96	34	115	81
10	21	126	77	88	88	97	29	81	88
11	24	127	76	78	78	98	32	82	78
12	24	124	73	71	72	97	32	83	73

*Note:* O, OMRON blood pressure meter; *N*, Nellcor Probe; *OEPS*, Opto-Electronic Patch Sensor.

The mean of the values measured from the commercial devices is 77.50 bpm, whereas the mean of the OEPS is 77.58 bpm, the difference of means is 0.08 bpm, the standard deviation (SD) is 2.27 bpm and the standard error of mean (SEM) is 0.65 bpm. Hence, the results from these tests show there is no difference in the SD as the SEM is very small.

**Figure 14:**
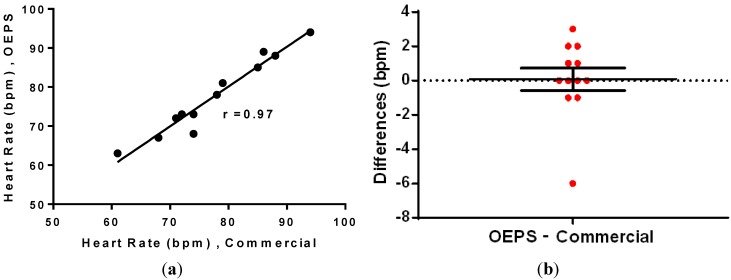
(**a**) Correlation; and (**b**) difference analysis of the HR data.

Additionally, the correlation and difference disruption were performed by using Wilcoxon test and it was found that the data is highly correlated, with a correlation coefficient *r* = 0.97 between the data, as shown in Figure 14a. In addition, the mean of differences was found to be 0.08, indicating that there is no significant difference between the data from OEPS and from the commercial monitor.

**Figure 15:**
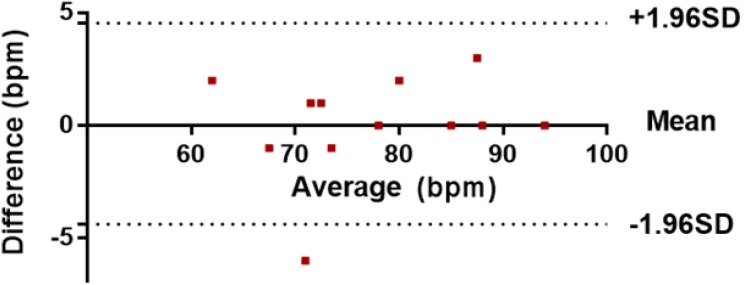
Bland-Altman comparative analysis of data.

The difference analysis in Figure 14b indicates a close correlation between sensors, with most of the values showing less than a *2* bpm difference. The correlation test also indicates effective performance, as the data is well correlated. Furthermore, Bland-Altman analysis was used to compare the measurements based on the difference *versus* the average of the two measurements, as shown in Figure 15.

Figure 15 shows a comparison of heart rate measurements from the OEPS and commercial devices, where each point represents a single instantaneous heart rate measurement (HR). The results out of this analysis show that the bias, which is the average of the differences, was equal to 0.08, and the 95% limits of agreement, which is a prediction band for the difference, were between 4.37 and 4.54. One value (−6) is out of the lower limit, as indicated by the fact that the scope of physical activity is beside the method of automatic motion artefact cancellation. The average of the differences (bias) is close to zero. If the bias were not close to zero, this would indicate that the two sets of data are producing different results.

**Figure 16:**
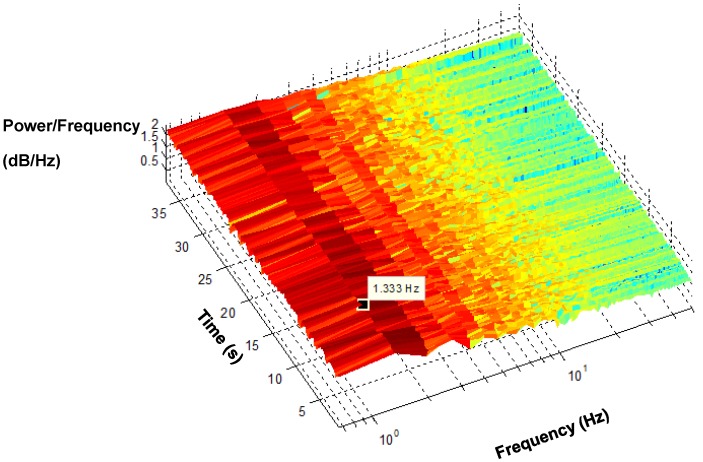
Short Time Fourier Transform (STFT) spectrogram of the PPG.

The spectrogram of a recovered PPG signal maps its energy content across frequency and time, as shown in Figure 16. It is difficult to obtain timing information from an FFT on the power spectrum. Hence, the spectrogram is a known method that provides time information along with frequency, which facilitated the tracking of how the frequency changes over time.

Figure 16 shows the dominant frequency components of PPG across the duration of time. The dark red indicates where the power of the signal is most significant, whereas yellow specifies less significant power. The dominant frequency originates from the PPG signals, appearing in the vicinity of 1.3 Hz.

## 4. Discussion 

In the present study, a quantitative and qualitative validation of the proposed AMC algorithm was performed using the corrupted PPG signals as a primary input and a motion reference signal that is generated from a 3MA. Despite the similarity between the results obtained from different axes, the implementation of a 3MA would be more advantageous than a single or two axes. For example, during sensor misalignment, a single vertical-axis could become misaligned, thus providing an ineffective noise reference signal. Hence, the 3MA signal would be a more effective noise reference. In contrast, since a single-axis represents only one physical plane of movement, only limited information would be acquired.

The recovery of useful physiological signals in this study has been achieved for motion along a particular axis, such *X, Y* and *Z*, and for fast walking in a random direction and cycling with resultant random motion. The vectors *X, Y* and *Z* representing motion acceleration are found to correlate during certain intervals with the corrupted signals captured by OEPS. The vector magnitude of three axes *X, Y* and *Z* was implemented to produce a motion reference signal that was used to reduce motion artefacts in the presence of corrupted PPG signals. The method was used especially for the components that share a long interval period between the corrupted signals and reference acceleration signals. However, it was found that some intervals were not well correlated with the motion reference; therefore, an adaptive filter was introduced and integrated with the initial cancellation stage to facilitate a better performance when recovering corrupted PPG signals, as shown in Figure 9 and Figure 10.

The results obtained from the processing algorithm are visible as it was tested in the frequency domain as shown in Figure 12 and Figure 16. Indeed, for resting conditions, where there is no significant movement, the PPG signals can be extracted very easily, as seen in Figure 7. However, in the presence of physical movement, the PPG signal is easily corrupted, as presented in Figure 6. Hence, the present approach was used in order to reduce the motion artefact through the use of the motion reference. To validate the data, several approaches were introduced, not only considering the frequency domain but also statistical tests, as shown in Figure 13, Figure 14 and Figure 15. The results support the present approach since a steady and reliable recovery of PPG signals was achieved under several experimental settings.

The algorithm of heart rate detection was executed on subject data acquired from the OEPS during exercise and comparing with reference heart rate measurements taken from commercial devices, namely a Nellcor probe and Omron blood pressure meter. When the fast walking speed was over >6 km/h, the comparison of HR between OEPS and Nellor probe is inadequate since the signals of the commercial probes were corrupted by motion artefacts. However the readings of heart rate (HR) were taken from other reference device at high speed, Omron blood pressure meter that can measure HR accurately. Finally, HR measurements of OEPS can be validated with clinical approved devices.

In addition, this study found that the amplitude of the signal can vary according to the measurement site as presented in Figure 7. The results found that the strongest amplitude from OEPS was achieved from the palm with green wavelength. The forehead and earlobe sites provided stable signals during walking; however, these signals had lower amplitudes in comparison to the palm. One interesting observation was that the lower illumination wavelength (525 nm) proved to be more stable than the red (650 nm) and infrared (870 nm) illumination wavelengths during walking and running. Under conditions of severe motion, for instance, running speeds >8.5km/h, the movement could cause the signal of the OEPS to saturate if the sensor was not properly attached and the signal could be corrupted; in these circumstances, signal reconstruction was not viable.

This approach was found to be promising since initial studies and preliminary results have included walking motions, as well as jogging, cycling and running activity, which is of particular interest since our goal is to develop a method for higher intensity exercise.

## 5. Conclusions

Opto-physiological centred measurement of human vital signs comprising a three-axis accelerometer could provide further opportunities for non-invasive and wearable biomedical monitoring *in vivo*. In the present research work, the cost-effective physiological measurement using OEPS demonstrates a viable concept for continuous and real-time wireless monitoring for personal healthcare, for instance the ageing population and for sufferers of chronic diseases.

An AMC method utilizing a 3MA to produce a reference signal demonstrates a good performance in the measurement of physiological parameters during regular body movements as described. The performance of the OEPS system using the AMC method depends on the assumption that the primary input of the corrupted signal will sometimes correlate with the reference signal captured simultaneously from the 3MA.

The approach presented in this study is capable of acquiring and processing physiological signals precisely and in real-time, as well as sending signals wirelessly to a monitor or server on the receiving end. The current findings add to our understanding of interaction of illumination and human tissue, and the correlation of the pulsatile waveforms with contributory noise effects on physiological measurements. The outcome from the present study is a further step towards the assembly of a practical patch sensor, and ultimately towards a cost-effective personal health tool that can be consolidated with available or emerging smart phones or similar devices. The technology also shows promise as a mobile and wearable clinical monitoring device that can overcome motion artefacts and provide an enhanced SNA. The work presents a high performance wearable OEPS that is viable and feasible for different daily activities, for instance, walking, jogging, cycling and running, with the prospect of becoming an effective tool, not only for personal health, but also for continuous sports and fitness monitoring.
